# Long-Stay Psychiatric Patients: A Prospective Study Revealing Persistent Antipsychotic-Induced Movement Disorder

**DOI:** 10.1371/journal.pone.0025588

**Published:** 2011-10-03

**Authors:** P. Roberto Bakker, Izaäk W. de Groot, Jim van Os, Peter N. van Harten

**Affiliations:** 1 Psychiatric Centre GGZ Centraal, Amersfoort, The Netherlands; 2 Dimence, Centre for Mental Health Care, Deventer, The Netherlands; 3 Department of Psychiatry and Psychology, South Limburg Mental Health Research and Teaching Network, EURON, Maastricht University, Maastricht, The Netherlands; 4 King's Health Partners, Department of Psychosis Studies, Institute of Psychiatry, King's College London, London, United Kingdom; Charité-Universitätsmedizin Berlin, Germany

## Abstract

**Objective:**

The purpose of this study was to assess the frequency of persistent drug-induced movement disorders namely, tardive dyskinesia (TD), parkinsonism, akathisia and tardive dystonia in a representative sample of long-stay patients with chronic severe mental illness.

**Method:**

Naturalistic study of 209, mainly white, antipsychotic-treated patients, mostly diagnosed with psychotic disorder. Of this group, the same rater examined 194 patients at least two times over a 4-year period, with a mean follow-up time of 1.1 years, with validated scales for TD, parkinsonism, akathisia, and tardive dystonia.

**Results:**

The frequencies of persistent movement disorders in the sample were 28.4% for TD, 56.2% for parkinsonism, 4.6% for akathisia and 5.7% for tardive dystonia. Two-thirds of the participants displayed at least one type of persistent movement disorder.

**Conclusions:**

Persistent movement disorder continues to be the norm for long-stay patients with chronic mental illness and long-term antipsychotic treatment. Measures are required to remedy this situation.

## Introduction

Antipsychotics remain the cornerstone of treatment in psychotic disorder. However, they may induce several side effects, one of which is movement disorder. Antipsychotic-induced movement disorder constitutes a major reason for non-compliance, resulting in an increased risk of psychotic relapse [1]–[3]. In addition, a meta-analysis [4] and two recent studies showed a higher mortality in patients with tardive dyskinesia (TD) [5], [6].

Antipsychotic-induced movement disorders [7], [8] can be divided in acute syndromes such as parkinsonism and akathisia, that occur within days or weeks after starting an antipsychotic, or after increasing the dose, and tardive syndromes, such as TD and tardive dystonia, that develop after months or years of antipsychotic treatment. In patients on long-term treatment with antipsychotics, combinations of acute and tardive syndromes may also occur.

Although second generation antipsychotics (SGAs) may be associated with a lower incidence rate of movement disorder, these medications nevertheless still carry risk [9]–[18]. In patients on long-term treatment with first generation antipsychotics (FGAs), the reported prevalence of antipsychotic-induced movement disorders was around 50 to 75% [19], [20]. Eleven long-term studies with SGAs (except clozapine) showed a reduced risk of drug-induced movement disorder, but not their expected disappearance [21]. These studies had several limitations such as lack of equivalent dosage of haloperidol in the control arm, high drop-out rates, short study duration and unreliable measurement of movement disorder. Three large, non-commercially funded trials published in the last five years found differences in the incidence of parkinsonism and akathisia, but no clear differences in the incidence of TD in a comparison between FGAs and SGAs (CATIE, Cutlass and EUFEST trial) [1], [9], [15], [17]. However, these studies also had methodological limitations such as a relatively short time to detect TD (around one year), high drop-out rates, and, in the Cutlass trial, many patients in the FGA group used sulpiride which has a lower incidence of movement disorder and is classified by some researchers as an SGA. A recent prospective cohort study with TD as primary outcome found no significant difference in the incidence of TD between patients taking FGAs and SGAs [22]. Leucht and colleagues [23] demonstrated that SGAs are a heterogenous group, each agent displaying its own particular properties. Furthermore, from a global perspective, the three antipsychotic drugs listed in the most recent (Index 2011) World Health Organization Model List of Essential Medicines are FGAs, namely chlorpromazine, fluphenazine and haloperidol (http://www.who.int/medicines/en).

Populations most at risk are those that are chronically exposed to antipsychotics, particularly when residing in hospital settings, where compliance likely is high and polypharmacy is common, further increasing risk for movement disorder [24]. Although long-stay settings are not mainstream, they remain a reality for a considerable number of patients with severe and chronic mental illness [25], and can be extended to the population in supervised residences in the community, where intake of medication often is similarly supervised. One retrospective survey reported existence of an antipsychotic polypharmacy regimen in 27.5% of the discharged patients with schizophrenia, such as concurrent use of FGAs and SGAs, in a tertiary psychiatric setting [26]. Broekema and colleagues [27] reported that the combination of SGAs and FGAs and/or anticholinergics constituted common practice in several European psychiatric hospitals. Routine cross-sectional data may not be suitable for the examination of rates of movement disorder in vulnerable populations with chronic mental illness, as drug-induced movement disorders fluctuate over time and remain underdiagnosed by both psychiatrists and neurologists [28]–[31].

For these reasons, a systematic and prospective assessment of movement disorder in a representative population of patients with long-term exposure to antipsychotics was used to examine the hypothesis that movement disorders remain highly prevalent in vulnerable populations.

## Methods

### Ethics statement

The protocol was approved by the standing Institutional Review Board, ‘Medisch-ethische Toetsingscommissie Instellingen Geestelijke Gezondheidszorg’ (Review Board for Human Research in Psychiatry), the Netherlands [protocol number 377].

Written informed consent was obtained from each patient; consent obtained from the next of kin was neither necessary nor recommended by the Review Board for Human Research in Psychiatry.

### Subjects

A 4-year prospective naturalistic study (July 2003–May 2007) was conducted in order to determine the frequency of TD, parkinsonism, akathisia and tardive dystonia in 209 patients with chronic mental illness. To this end, a cohort was drawn from a general psychiatric hospital (GGZ Centraal, Amersfoort, the Netherlands). Inclusion criteria were: minimum age of 18 years and cumulative exposure to antipsychotics for at least 1 year. Exclusion criteria were: history of neurological disorders impacting on motor function. The cohort was representative of the population of patients with the most severe chronic mental illness requiring long-stay care, given that the hospital serves an epidemiological catchment area, is the only institute providing this type of care and patients were approached using a comprehensive list of all in-patient.

Of the patients assessed at baseline (N = 207) 93.7% (n = 194) had one follow-up and 59.4% (n = 123) had two follow-up assessments. Loss to follow-up was due to patients who were difficult to trace after leaving hospital, as well as patients dying or patients refusing assessment after inclusion.

### Assessment

Patients were examined by a trained psychiatrist (PRB), using a standard protocol, described by van Harten and colleagues [32]. Patients were barefooted and seated in a chair without armrests. The researcher asked detailed questions about (i) use of chewing gum or candy at the moment of assessment as well as (ill-fitting) dentures, as both may be misdiagnosed as orofacial movement disorders, and (ii) subjective akathisia. The patient performed different tasks to assess the existence of movement disorders and to provoke abnormal movements. Thus, the following positions were adopted in succession: resting arms on the lap in different positions, arms hanging aside, stretching arms, making fast alternating hand and foot movements, opening the mouth, showing the tongue, rising from chair, and walking. Additionally, posture, rigidity and balance were assessed. Tongue dyskinesia was provoked by fingertip movements, and objective akathisia by talking conversationally while the patient was standing.

Originally, in addition to the term ‘acute’, the term ‘tardive’ (delayed) was introduced to emphasize the late-onset types of movement disorders during antipsychotic use. Yet, the definition in the current study emphasizes their persistence, which is more important [8], [33].

Dyskinesia [34] was defined as hyperkinetic choreiform involuntary movements which often fluctuates in severity. TD was assessed with the Abnormal Involuntary Movement Scale (AIMS) [35], [36] and case definition was based on Schooler and Kane criteria [37], requiring (i) the presence of moderate dyskinesia in at least one body area or mild dyskinesia in at least two body parts, and (ii) the absence of other conditions resulting in abnormal involuntary movements.

Parkinsonism was assessed with the Unified Parkinson Disease Rating Scale (UPDRS) [38]. A case definition of parkinsonism was based on (i) ‘mild’ expression of rest-tremor or rigidity as both are typical of parkinsonism, and (ii) if no tremor or rigidity was rated, the cut-off point was one rating of ‘moderate’ or two ratings of ‘mild’ on items of bradykinesia and postural stability. The more stringent criteria for items of bradykinesia and postural stability were chosen as these symptoms may be part of psychiatric syndromes or sedation. Besides this definition, an additional case definition of parkinsonism was applied in accordance with the UK Brain Bank definition, using a score of 2 in the bradykinesia items of the motor UPDRS, and a score of 1 in the items rest tremor, rigidity or postural instability of the motor UPDRS.

Akathisia [8] was defined as both subjective inner feelings of restlessness and objective motor (leg) movements. A case definition of akathisia was based on a rating of at least ‘mild’ on the global akathisia item. Akathisia was assessed with the Barnes Akathisia Rating Scale comprising an objective and a subjective item [39].

Dystonia was defined as a syndrome of sustained muscle contraction, frequently causing twisting and repetitive movements or abnormal postures [40]. Tardive dystonia was diagnosed, following Burke's criteria [41], if one body area attracted a rating of at least ‘mild’ or if two or more body areas attracted a rating of ‘slight’ on the Fahn-Marsden scale [42]. As frequent eye-blinking (rating of ‘mild’ on the item ‘eye’) has many causes, case definition of tardive dystonia required a rating of at least ‘moderate’ (blepharospasm) when ‘eye’ was the only symptom area.

The case definition of a persistent movement disorder was based on 2 consecutive assessments over a period of minimally 3 months, and required that individuals met case definition criteria at two consecutive assessments (hereafter: persistent movement disorder).

Guided by previous literature, variables possibly affecting risk were extracted from patients' case notes including age, sex, diagnosis according to DSM-IV, ethnic group (classified as white and non-white) and duration of hospitalization. At baseline and at each follow-up assessment, current use of antipsychotic and anticholinergic medication was collected from the hospital and outpatient pharmacy databases.

The diagnosis ‘schizophrenia’ hereafter refers to DSM-IV codes 295.30, 295.10, 295.20, 295.90, 295.60, 295.70, and other diagnoses of ‘psychotic disorder’ to 295.40, 297.1, 298.8, 298.9.

### Statistical Analyses

Frequency of persistent movement disorder was calculated in patients with minimally two assessments. Chi-squared tests and nonparametric trend tests were applied to categorical data.

Antipsychotic doses were converted to defined daily dose (DDD), assigned and reviewed by researchers of the World Health Organisation Centre of Drug Statistics Methodology (WHO, *Collaborating Centre for Drugs Statistics Methodology Available at:* http://www.whocc.no/atcddd/ *. Accessed* December 2010). DDD was chosen as it better reflects the observed multireceptor involvement of antipsychotics, unlike classic chlorpromazine (CPZ) equivalents which are based mainly on dopamine-2 receptor occupancy. In addition, DDD equivalents are updated periodically. Anticholinergic medication was modeled as a dichotomous variable (yes/no).

## Results

### Sample Characteristics

Of the 209 patients included, one patient developed a brain tumor, another patient died after inclusion. All patients had a history of cumulative antipsychotic intake of minimally 1 year. Attrition was 9.8%.

Most patients were white (85.0%) and had chronic mental illness requiring long-term admission. At baseline, the mean (SD) age was 47.4 (12.8) years; men 46.3 (12.8) and women 49.1 (12.7) of age. The mean (SD) age at first admission was 25.0 (8.4) years; men 23.8 (7.6) and women 26.7 (9.3) of age at first admission. The total duration of admission was 22.1 (13.1) years. Diagnoses according to DSM-IV Axis I as defined above were: schizophrenia 69.6%, psychosis 5.3%, affective disorder 13.5%, other Axis I diagnosis 6.8% and no Axis I (with a Axis II) diagnosis 4.8%.

At baseline and follow-up, antipsychotics were used by 89.3–98.5% of the patients; FGA and SGA in 64.8–67.5% and 55.7–61.3%, respectively; FGA only and SGA only in 33.0–37.3% and 24.6–32.8%, respectively; 28.4% used both FGA and SGA at baseline; use of 0, 1, 2, 3 and 4 antipsychotic(s) was observed in 1.5–10.7%, 41.9–55.4%, 34.3–40.8%, 4.1–8.3% and 0.5–1.6%, respectively; total DDD equivalent antipsychotic use was 2.3–2.5.

### Frequency over period of observation

Over the period of observation (mean = 1.1 years, SD = 0.64), at baseline and follow-up, the frequencies of movement disorder in the sample were 30.4–36.6% for TD, 21.7–32.5% for orofacial TD, 11.9–13.9% for limb truncal TD, 62.9–65.9% for parkinsonism, 13.8–26.3% for rest tremor, 6.6–15.0% for rigidity, 53.6–61.0% for bradykinesia, 8.8–10.4% for akathisia and 8.1–16.0% for dystonia. The frequency of persistent movement disorder in the sample was 28.4% for TD, 20.1% for orofacial TD, 7.7% for limb truncal TD, 56.2% for parkinsonism, 12.9% for rest tremor, 6.7% for rigidity, 48.5% for bradykinesia, 4.6% for akathisia and 5.7% for dystonia. Sixty-eight percent of the participants had at least one type of persistent movement disorder, 43.3% had a single type of persistent movement disorder, and 24.7% had at least 2 types of persistent movement disorder (Table 1). Using the UK Brain Bank definition, the frequencies of parkinsonism were 51.2–60.3% at baseline and follow-up, whereas the frequency of persistent parkinsonism was 53.1%.

**Table 1:** Period frequency^a^ of persistent drug-induced movement disorders^b^^,^ c (N = 194, men = 114, women = 80).

Movement disorder	N	%
Tardive dyskinesia	55	28.4
Orofacial TD^d^	39	20.1
Limb truncal TD	15	7.7
Parkinsonism	109	56.2
Rest tremor	25	12.9
Rigidity	13	6.7
Bradykinesia	94	48.5
Akathisia	9	4.6
Tardive dystonia	11	5.7

a Mean period was 1.1 year (SD 0.6).

b Persistent movement disorder: 2 consecutive positive assessments with an interval of at least 3 months.

c 132 (68.0%) had at least one type of movement disorder.

d Tardive dyskinesia.

Table 2 shows the frequency of persistent movement disorder, by age group defined by the tertile cut-offs of the age distribution. In the nonparametric test for trend, frequency of persistent TD, parkinsonism and tardive dystonia increased with increasing age (p = 0.005, p = 0.000 and p = 0.06, respectively). Frequency of persistent akathisia decreased significantly with increasing age (p = 0.039), such that the age group of 53 and older did not display any akathisia. Frequency of persistent parkinsonism in accordance with UK Brain Bank definition, by age group, was 32.3%, 56.9% and 70.3%, respectively (p = 0.000).

**Table 2:** Period frequency^a^ of persistent drug-induced movement disorder^b^ in 194 patients, by tertile age group.

	Age (years)^c^				
Movement disorder (%)	≤40 (n = 65)	41–52 (n = 65)	≥53 (n = 64)	z^c^	p
Tardive dyskinesia (n = 55)	15.4	32.3	37.5	2.78	0.005
Parkinsonism (n = 109)	40.0	49.2	79.7	4.52	0.000
Akathisia (n = 9)	7.7	6.2	0.0	−2.07	0.039
Tardive dystonia (n = 11)	1.5	6.2	9.4	1.92	0.055

a Mean period was 1.1 year (SD 0.6).

b Persistent movement disorder: 2 consecutive positive assessments with an interval of at least 3 months.

c Nonparametric test for trend across ordered groups (extension of the Wilcoxon rank-sum test).

Frequency of persistent TD, parkinsonism, akathisia and tardive dystonia did not differ between FGA only and SGA only, both at baseline and at follow-up (p-values 0.506–0.898, 0.392–0.962, 0.184–0.576 and 0.424–0.916, respectively). Parkinsonism in accordance with UK Brain Bank definition did not differ between FGA only and SGA only, both at baseline and at follow-up (p-values 0.705–0.929).

## Discussion

This study showed that persistent movement disorder remains highly prevalent in long-stay patients with chronic mental illness and long-term antipsychotic treatment. The high period frequency of 68% with at least a single drug-induced movement disorder is even more striking given the use of strict case definition criteria that had to be positive on at least two consecutive assessments. Clinical relevance of these findings is suggested not only because of the high frequency of these acute and tardive movement disorders, but also because persistence of movement disorder seems to be the rule. This implies that most patients on long-term antipsychotic treatment have persistent movement disorder which make this side effect a matter of urgent consideration.

Frequencies of TD, parkinsonism and dystonia were associated with older age, albeit the latter at trend significance only. In contrast, akathisia was negatively associated with older age, and even completely absent in the oldest age group. This observation could not be explained by dosage as a *post-hoc* analysis showed that total DDD equivalent at baseline and follow-up moments were neither strongly nor significantly associated with age (r = −0.02, p = 0.76; r = −0.13, p = 0.08; r = −0.10, p = 0.27, respectively). Furthermore, around 50% of the patients used more than one type of antipsychotic with a DDD equivalent above 2.3. This is a considerable high antipsychotic dosage, as the DDD is the assumed average daily dose for a drug used for its core [43]. Yet, frequency of movement disorder between FGA and SGA did not differ.

We compared frequencies of parkinsonism between the UK Brain Bank definition and ours, and found similar results at baseline and follow-up; the same held for persistent parkinsonism.

### Limitations

First, it may be hypothesized that the varying number of follow-up assessments (from 1 to 2) in the participants may have contributed to an unstable estimate. However, frequency of persistent movement disorders in those with 1 and 2 follow-up assessments were similar (data not shown). Second, the cohort in the current study was representative of the population of patients with the most severe chronic mental illness requiring long-stay care, the target population for this study. Thus, results cannot be extrapolated to the entire population of psychiatric patients exposed to antipsychotics, in whom rates of movement disorder may be different. Third, in the current study, the mean follow-up time seemed sufficient (1.1 years) to detect a persistent movement disorder because the patients were on long-term antipsychotic treatment, i.e., were exposed for a sufficiently long period to develop a persistent movement disorder. Although this study cannot draw firm conclusions regarding the persistence of movement disorders in the long run, most long-term follow-up studies nevertheless report high persistence rates. Fourth, the classic model of movement disorders originating from antipsychotics is challenged by a large body of literature and two meta-analyses [44], [45] demonstrating higher prevalence rates of movement disorders in patients with a diagnosis of schizophrenia. These results provide a strong argument for the hypothesis that movement disorders may not exclusively result from antipsychotic treatment but also reflect a fundamental aspect of neurodevelopmental pathophysiology involving sensitization of dopaminergic nigrostriatal circuits [46]–[49]. There is no phenomenological difference between parkinsonism and dyskinesia related to schizophrenia versus drug-induced parkinsonism and dyskinesia. As a consequence, caution is required in interpreting the findings. Future prospective studies in populations of drug-naive patients with a first episode of psychosis before and after antipsychotic treatment are essential to make a distinction between primary (part of schizophrenia) and secondary (drug-induced) movement disorder. Even so, primary symptoms may develop in the course of schizophrenia making differentiation between primary and secondary symptoms difficult.

Although it is not possible to differentiate between primary and secondary movement disorders in long-stay patients, and the two types likely often occur in combination, distinguishing between the two types is of little consequence for treatment interventions which often consist of lowering the dosage of the antipsychotic, switching to an SGA (preferably clozapine), or adding an anticholinergic.

### Strengths

First, all assessments were performed by a single person, who was trained and retrained (in order to prevent ‘drift’) regularly by the senior author (PNvH), an expert in the assessment and diagnosis of movement disorders. Second, a naturalistic and pragmatic design was used in a representative chronic psychiatric population, reflecting real-life clinical practice [50], and therefore yielding high external validity. Third, definition of persistent movement disorder was based on 2 consecutive assessments over a period of minimally 3 months, which is in contrast with many previous studies in which case definition was defined cross-sectionally. Persistent movement disorder may be a more valid measure, as it more specifically defines the disorder category given the continuously fluctuating nature of the phenotypes under investigation.

The prevalence of movement disorder from previous studies, as mentioned below, concur with the current study for TD, but they tend to be lower for parkinsonism, and tend to be higher for akathisia as well as for tardive dystonia. However, previous studies do not match with the current study, given the fact that these used cross-sectional measures and did not focus on the vulnerable subgroup of long-stay patients in hospital.

### Tardive dyskinesia

Reported prevalence rates of TD vary from 3% to 70% with a median rate of 24%, most of the TD being mild, with higher rates in the elderly [51]. Van Harten and colleagues [32] reported a TD prevalence of 39.7%. A recent meta-analysis concluded that age was a likely, although not quite conclusive, risk factor for TD [52]. Other risk factors have been suggested, but with little meta-analytic support [52].

### Parkinsonism

In the study by Modestin and colleagues [47] the prevalence of parkinsonism in 1995 and 2003/4 was 17% and 29%, respectively. Janno and colleagues [19] estimated the prevalence of parkinsonism at 23.2% and 72.7%, according to DSM-IV criteria and Simpson-Angus Scale criteria, respectively. Van Harten and colleagues [32] reported a parkinsonism prevalence of 36.1%. Older age may be a risk factors for parkinsonism [7], but other studies showed a higher risk in younger patients [53], [54].

### Akathisia

Modestin and colleagues [47] reported a 14% prevalence rate of akathisia that was constant over two time points. Janno and colleagues [19] reported prevalence rates of 31.3% and 27.3%, according to DSM-IV criteria and the Barnes scale, respectively. In the study by van Harten and colleagues [32] the reported prevalence of akathisia was 9.3%. In two retrospective studies in younger patients, neither age nor sex was related to tardive akathisia [55]. In another study, particularly younger patients taking higher dosage of (depot) antipsychotics were at risk of chronic akathisia [56]. In addition, prevalence of akathisia showed a decreasing trend with age [32].

### Tardive dystonia

Van Harten and Kahn [40], reviewing 13 studies, calculated a mean prevalence of tardive dystonia of 5.3%. Earlier studies tended to show lower prevalence rates for tardive dystonia than later ones, probably owing to respectively higher and lower thresholds used, and to differences in rating scales. Van Harten and colleagues [32] reported a high prevalence (13.4%) of tardive dystonia; the high rate was thought to relate to the fact that the group examined was black and/or the fact that a careful standard examination with two investigators with a comprehensive rating scale was applied. Other studies reported comparably high prevelances of 11% [57] and 21.6% [58]. Tardive dystonia is evenly distributed across the age of onset range from 13 to 72 years, and tends to generalize in younger patients [8]. Patients developing dystonia in isolation tend to be younger than those with ‘classical’ TD [7].

Van Harten and colleagues [32] found a high prevalence of one or more types of movement disorders (73.7%). Furthermore, in the study by Janno and colleagues [19], 61.6% of the patients had at least one movement disorder according to DSM-IV criteria.

Having persistent drug-induced movement disorders seems to be the norm for long-stay patients with chronic mental illness and long-term antipsychotic treatment. We were surprised by the few notes about these side effects in the files of the patients, which has been found by others also [28]–[31]. The relative lack of focus on movement disorder syndromes is reflected in the very low rate of DSM-IV axis I diagnosis of these in routine clinical practice. Several reasons may be responsible for this discrepancy between clinical reality and clinical attention. First, it is not common practice to do a systematic investigation toward drug-induced movement disorders, which will limit recognition. Second, clinicians may wrongly assume that drug-induced movement disorders are almost not treatable. In fact, the interventions to prevent or treat akathisia and parkinsonism are evidence based and are quite easy to implement in clinical practice. Although suggested strategies to prevent/treat TD [59] or tardive dystonia [7] are not evidence-based, they resemble the strategies used to prevent acute movement disorders. In addition, novel treatment options are being developed, such as botulinum toxin, tetrabenazine, branched-chain amino acids, and, in very severe cases, deep brain stimulation [60]–[64]. Third, the introduction of the SGAs led to the expectation that drug-induced movement disorders would disappear but they only reduce the risk. Furthermore, antipsychotics are increasingly used for other indications as SGAs have strong mood stabilizing properties which will increase the absolute numbers of drug-induced movement disorders. Fourth, most patients with schizophrenia do not complain of their movement disorder [65]–[67]. Unawareness of movement disorder and subsequent lack of subjective complaints is a risk factor for diagnostic delay [66]. In addition, the unawareness notwithstanding, a movement disorder has a stigmatizing effect on patients and a negative effect on quality of life. Therefore, active assessment and treatment of movement disorder, similar to the current increased focus on metabolic syndrome, is of paramount importance. Owens [7] stated that movement disorder now can be seen as a quality-of-care-issue. In addition, shared care decision making and informed consent is part of antipsychotic treatment [68]. Systematic diagnosis may help physicians become more aware of movement disorders.

In conclusion, persistent movement disorder continues to be the norm for long-stay patients with chronic mental illness requiring long-term antipsychotic treatment, and therefore measures are required to remedy this situation, making it part of routine quality-control procedures. It may be considered somewhat ironic that long-stay patients with chronic mental illness pay a high price for the intensive care they receive, particularly because effects are likely mediated by the relatively high compliance with pharmacotherapy in these settings. Although long-stay settings are not present in abundance anymore, they are also not rare. In the U.S., over 200 state hospitals attend a declining but challenging patient population [69] and the findings likely can be extended to the considerably larger group of patients who live in supervised residential settings. Systemic screening for movement disorder takes little time and can be easily implemented in clinical practice. In addition, given the clear age dependency of some movement disorders, elderly patients are a group of special concern.
